# Correia Repeat Enclosed Elements and Non-Coding RNAs in the *Neisseria* Species

**DOI:** 10.3390/microorganisms4030031

**Published:** 2016-08-25

**Authors:** Sabrina B. Roberts, Russell Spencer-Smith, Mahwish Shah, Jean-Christophe Nebel, Richard T. Cook, Lori A. S. Snyder

**Affiliations:** 1 School of Life Sciences, Pharmacy, and Chemistry, Kingston University, Penrhyn Road, Kingston upon Thames, KT1 2EE, UK; sabrina.b.roberts@hotmail.com (S.B.R.); rssmith151@gmail.com (R.S.-S.); mahwishshah@hotmail.co.uk (M.S.); r.cook@kingston.ac.uk (R.T.C.); 2 Department of Pharmacology, University of Illinois at Chicago, Chicago, IL 60612, USA; 3 School of Computing and Information Systems, Kingston University, Penrhyn Road, Kingston upon Thames, KT1 2EE, UK; j.nebel@kingston.ac.uk

**Keywords:** *Neisseria gonorrhoeae*, *Neisseria meningitidis*, CREE, ncRNA, J0101

## Abstract

*Neisseria gonorrhoeae* is capable of causing gonorrhoea and more complex diseases in the human host. *Neisseria meningitidis* is a closely related pathogen that shares many of the same genomic features and virulence factors, but causes the life threatening diseases meningococcal meningitis and septicaemia. The importance of non-coding RNAs in gene regulation has become increasingly evident having been demonstrated to be involved in regulons responsible for iron acquisition, antigenic variation, and virulence. *Neisseria* spp. contain an IS-like element, the Correia Repeat Enclosed Element, which has been predicted to be mobile within the genomes or to have been in the past. This repeat, present in over 100 copies in the genome, has the ability to alter gene expression and regulation in several ways. We reveal here that Correia Repeat Enclosed Elements tend to be near non-coding RNAs in the *Neisseria* spp., especially *N. gonorrhoeae*. These results suggest that Correia Repeat Enclosed Elements may have disrupted ancestral regulatory networks not just through their influence on regulatory proteins but also for non-coding RNAs.

## 1. Introduction

Gonorrhoea poses a global threat that may become virtually untreatable due to antibiotic resistance, including increasing prevalence of azithromycin-resistant isolates [1,2,3,4] resulting in unsuccessful first-line dual treatment with ceftriaxone and azithromycin. Treatment for meningococcal meningitis and septicaemia must be rapid and effective or the infection will be fatal [5,6,7,8]. Concerns over the growing problem of antibiotic resistance in several bacterial species have driven investigations into RNA-based therapeutics to manipulate bacterial gene expression [9,10].

Non-coding RNAs (ncRNAs) can be important regulators of gene expression with roles in both physiology and disease [11,12]. Small ncRNAs have been shown to be involved in regulatory networks and gene expression in the *Neisseria* spp. [13,14,15,16,17,18,19,20].

Small ncRNA *aniS* was annotated as a coding sequence (NMB1205 in the meningococcus and NGO0796 in the gonococcus), leading to its inclusion in the design of microarrays that have shown its differential expression [13,21,22,23,24,25,26]. The ncRNA *nrrF* has been shown to be part of the *fur* regulon [13,16]. RNA-seq, using next-generation sequencing, has identified several potential ncRNAs due to the depth and specificity of transcript data that can be identified [17,20,27]. As a result, a ncRNA adjacent to pilin gene *pilE* was identified and shown to be required for pilin antigenic variation [14,19]. Deletion of selected small ncRNAs decreases the virulence of *Neisseria meningitidis* in the rat model [15]. Eleven of the identified and validated ncRNAs were highly conserved, three were associated with transposable elements, and five were absent from the non-pathogenic *Neisseria lactamica*. RNA-seq and transposon insertion site sequence mapping identified 253 ncRNAs in *Neisseria gonorrhoeae* [28]; 59 are intergenic and nine were validated by Northern blotting. Three new ncRNA transcripts were confirmed in the *Neisseria gonorrhoeae* MS11 strain.

Other non-coding features of the *Neisseria* spp. genomes are the Correia Repeat Enclosed Elements (CREE) [29]. A *N. gonorrhoeae* genome contains just over 100 copies [30] of the CREE [30]. *N. meningitidis* has roughly twice as many CREE within its genome [29,31]. Correia repeats were originally identified as 26 bp sequences often present as inverted repeats [32,33]. CREE are 69–151 bp regions with the Correia inverted repeat and a characteristic core region. These are found most commonly in intergenic regions and are often near virulence, metabolic, and transporter genes [29].

This study investigated whether CREE are located near predicted small ncRNAs in *N. gonorrhoeae*, *N. meningitidis*, and *N. lactamica*. RNA-seq was used to support ncRNA predictions.

## 2. Materials and Methods

Candidate small ncRNAs were identified using SIPHT [34]. GenBank entries for each of the eight *Neisseria* spp. investigated here were downloaded on 24 October 2012, accession numbers: *N. gonorrhoeae* strain NCCP11945 (NC_011035); *N. gonorrhoeae* strain FA1090 (NC_002946); *N. meningitidis* strain MC58 (NC_003112); *N. meningitidis* strain Z2491 (NC_003116); *N. meningitidis* strain FAM18 (NC_008767); *N. meningitidis* strain alpha14 (NC_013016); *N. meningitidis* strain 53442 (NC_010120); *N. lactamica* strain 020-06 (NC_014752). SIPHT settings used: MaxE 5.00E-3; minimum length 30; maximum length 550; minimum TT confidence 86; and maximum RNAMotif score −6. These are the default settings when a new analysis is launched on the SIPHT web interface (http://newbio.cs.wisc.edu/sRNA/apps/sRNA/submitjob_pegasus_web.php).

The locations of the CREE within *N. gonorrhoeae* strains FA1090 and NCCP11945 were reported previously [30]. Within *N. meningitidis* strains MC58, Z2491, FAM18, alpha14, and 053442, and *N. lactamica* strain 020-06, CREE were identified using the Fuzznuc pattern finder [35] within *x*BASE [36], as had been done previously for the gonococci [30]. Search terms were based on the previously reported variants of the 26 bp inverted Correia repeat: 5′-tatagtggattaacaaaaaccggtacgg-3′; 5′-tatagtggattaaatttaaaccggtacgg-3′; 5′-tatagtggattaacaaaaatcaggacaa-3′; 5′-tatagtggattaaatttaaatcaggacaa-3′ [30,32,33].

CREE and ncRNA location data was organised in Excel matching CREE with the closest ncRNA for *N. gonorrhoeae* strain NCCP11945. Statistical analyses used the Kolmogorov-Smirnov test and non-parametric Spearman’s Rho correlation analysis in IBM SPSS Statistics version 21 to assess the distribution of CREE in the genome and between the CREE and ncRNAs.

*N. gonorrhoeae* strain NCCP11945 was grown on GC agar (Oxoid) at 37°C, 5% CO_2_ overnight. Growth was immediately removed to RNA*Later* (LifeTechnologies) and RNA extracted using the RNasy kit (Qiagen). RNA quality was determined on the 2100 Bioanalyzer (Agilent) to have a RIN of 9 or above. RNA-seq used the Ion Personal Genome Machine, Ion Total RNA-Seq Kit with ERCC RNA Spike-In Control Mix, Ion Express Template kit, Ion Sequencing kit (Life Technologies), and 1 μg of rRNA-depleted RNA. RNA-seq data (GEO accession numbers GSE58650 and GSE73032) was aligned against the reference genome NCCP11945 using NextGENe v2.3.4.2.

## 3. Results

Small ncRNAs were predicted using SIPHT [34] for *N. gonorrhoeae* strains NCCP11945 and FA1090, *N. meningitidis* strains MC58, Z2491, FAM18, alpha14, and 053442, and *N. lactamica* strain 020-06 (Table S1 to Table S8). Between 760 and 996 ncRNAs were predicted to be present in these *Neisseria* spp. genomes (Table 1, Table S1 to Table S8). These results include the known ncRNA *nrrF* [13,16] (Table S4, Candidate 223–225), the ncRNA adjacent to *pilE* [14,19] (Table S1, Candidate 468; Table S4, Candidate 182; Table S6, Candidate 880), and *aniS* [24] (Table S1, Candidate 793; Table S6, Candidate 716;). The *aniS* sequence is annotated as a putative protein encoding gene in strains FA1090 and MC58 (NGO0796 and NMB1205, respectively). SIPHT uses only intergenic regions for its predictions, therefore the *aniS* annotation as a CDS excludes it from detection by SIPHT. Annotations as CDSs may account for otherwise conserved ncRNAs being absent from some SIPHT predictions.

CREE locations were previously reported for *N. gonorrhoeae* strains NCCP11945 and FA1090 [30] (Table 2; Table S9 and Table S10). CREE locations were identified here using the same method for *N. meningitidis* strains MC58, Z2491, FAM18, alpha14, 53442, and *N. lactamica* strain 020-05 (Table 2; Table S11 to Table S16). On average, 127 CREE were identified in *N. gonorrhoeae* and 249 in *N. meningitidis*. In the single *N. lactamica* strain 92 were found, similar to previous analyses of non-pathogenic *Neisseria* spp. [37,38].

The output of SIPHT was combined with the CREE location information for each genome and those within 1000 bp were identified (Table S17 to Table S24). Starting from the distance of 1000 bp, the distances between the CREE and ncRNAs were assessed. This revealed that most of the CREE were within 300 bp of a predicted ncRNA (Table 3). For example, in strain NCCP11945 86 of the 131 CREE are within 300 bp of a predicted ncRNA (66%) (Table 3; Table S17). There are fewer CREE within 300 bp of a predicted ncRNA in the meningococci (average 56%) than in the gonococci (average 71%) and *N. lactamica* (64%) (Table 3; Table S17 to Table S24).

Many of the CREE overlap the sequences of predicted ncRNAs (Table 3; Table S17 to Table S24). In gonococcal strain NCCP11945, for example, 57% of all of the CREE (75 out of 131) in the genome overlap with predicted ncRNAs (Table 3; Table S17). There are fewer overlaps seen in *N. meningitidis* and *N. lactamica*, where on average 37% and 39%, respectively, of all CREE overlap with predicted ncRNAs, compared to a 58% average in *N. gonorrhoeae* (Table 3).

**Figure 1:**
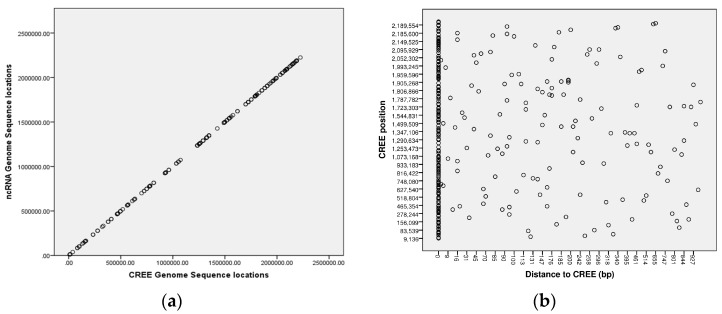
Statistical analysis of the associations between CREE and predicted ncRNAs. (**a**) Using SPSS, CREE locations in *N. gonorrhoeae* strain NCCP11945 were tested for a normal distribution using the Kolmogorov-Smirnov test. Non-parametric Spearman’s Rho correlation analysis was employed to determine correlation between CREE locations (*x*-axis) and the SIPHT predicted ncRNA genome sequence locations (*y*-axis). (**b**) Statistical analysis of the start/end points of the CREE and the start/end points of the SIPHT predicted ncRNAs shows a close association of these elements in the chromosome. On the *x*-axis is plotted the distance of the ncRNA to the CREE in basepairs, while on the *y*-axis are the CREE positions along the *N. gonorrhoeae* strain NCCP11945 chromosome. This shows that the majority of the CREE are overlapping ncRNAs.

Using all of the CREE start or end locations for strain NCCP11945 matched to the nearest corresponding ncRNA start or end locations (Table S25), the Kolmogorov- Smirnov test indicated that the CREE locations were not normally distributed (*p* < 0.001). Using the same test, CREE distribution around the genome was shown to be neither random (*Z* = 5.292; *p* < 0.001) nor uniform (*Z* = 1.557; *p* = 0.016). Spearman’s Rho correlation demonstrated that CREE locations were very strongly correlated with ncRNA locations (ρ = 1; *p* < 0.001). The ncRNA locations were all either located within or very close to CREE regions (Figure 1).

Alignment of RNA-seq data against the *N. gonorrhoeae* strain NCCP11945 genome sequence showed ncRNA transcription (Table S26) at 91 out of 96 locations (95%) where CREE are near to the ncRNAs (Table 3). Read coverage varied: ncRNAs with ≤10 reads were considered low coverage (74 of 91, 81%); >10 to ≤25 were considered medium coverage (3 of 91, 3%); and >25 were considered high coverage (14 of 91, 15%) (Table S26). Genes at high coverage locations included virulence associated genes *pilQ*, pilin, outer membrane genes, as well as chaperone related genes (Table S27).

## 4. Discussion

SIPHT predicts between 760 and 996 ncRNAs in the *Neisseria* spp. genomes investigated present within the 1782 to 2255 intergenic regions (Table 1; Table S1 to Table S8). Amongst these predictions are several previously reported and verified ncRNAs [13,14,16,19,24,25,39], which supports the accuracy of the SIPHT predictions. The predictions reported here include some that span the same region of sequence, where only one is likely to be a ncRNA, and some that may be over-predictions. However, these predictions provide a starting point for investigations into ncRNAs in these species.

Correia Repeat Enclosed Elements are unique to the *Neisseria* spp. [29,30]. They have been demonstrated to insertionally inactivate genes [31] and to disrupt ancestral regulatory systems through CREE-associated promoters [40]. There are, on average 127 CREE in a *N. gonorrhoeae* genome, 249 in a *N. meningitidis* genome, and there are 92 CREE in the *N. lactamica* strain 050-20 (Table 2; Table S9 to Table S16).

CREE tend to be located within 300 bp of predicted ncRNAs, with many overlapping (Table 3; Table S17 to Table S25). The frequency with which CREE overlap predicted ncRNAs is higher in *N. gonorrhoeae* (average 58%) than in *N. meningitidis* (average 37%) and *N. lactamica* (39%). This is particularly of note because there are only half as many CREE in *N. gonorrhoeae*, yet those that are present are far more likely to be located close to ncRNAs and to overlap the ncRNA sequences. In *N. lactamica*, the number of CREE in the genome is closer to that seen in the gonococcus and yet the overlaps between CREE and ncRNAs are on par with *N. meningitidis* (Table 3). Given the known roles of CREE in gene regulation [40,41,42], it is possible that the presence of CREE and its associated promoters may influence the expression of small ncRNAs through insertional inactivation and/or disruption of ancestral regulatory networks through introduction of CREE-associated promoters.

RNA-seq data supports the SIPHT predictions (Table S26), demonstrating transcription of 95% of the ncRNAs that have adjacent or overlapping CREE in *N. gonorrhoeae* strain NCCP11945. Those with the highest detected transcription are upstream of virulence determinants (Table S27), further supporting the important role of ncRNAs in these pathogens.

## 5. Conclusions

In the *Neisseria* spp., CREE are frequently found near or overlapping ncRNAs in the genome. CREE may influence the expression of ncRNAs through the presence of their promoters and/or insertional activation, similar to the role of CREE in gene regulation. It may be possible to exploit differences between the species with regards to ncRNAs and their interaction with CREE in the design of RNA-based therapies to restrict the meningococcus to the mucosal surface, where it is as harmless as *N. lactamica*, and to prevent gonococcal antigenic variation, enabling rapid clearance and immunity.

## Figures and Tables

**Table 1:** Number of intergenic regions and predicted non-coding RNAs (ncRNAs) in *Neisseria* spp. genome sequences.

Strain	Intergenic Regions	SIPHT ncRNAs ^1^
*N. gonorrhoeae* strain NCCP11945	2255	760
*N. gonorrhoeae* strain FA1090	1806	976
*N. meningitidis* strain MC58	2015	912
*N. meningitidis* strain Z2491	1846	996
*N. meningitidis* strain FAM18	1833	959
*N. meningitidis* strain alpha14	1782	976
*N. meningitidis* strain 53442	1881	846
*N. lactamica* strain 020-06	1873	890

^1^ Number of SIPHT predicted small non-coding RNAs in the genomes (Table S1 to Table S8).

**Table 2:** Number of Correia Repeat Enclosed Elements in *Neisseria* spp. genome sequences.

Strain	Intergenic Regions	CREE ^1^
*N. gonorrhoeae* strain NCCP11945	2255	131
*N. gonorrhoeae* strain FA1090	1806	123
*N. meningitidis* strain MC58	2015	248
*N. meningitidis* strain Z2491	1846	260
*N. meningitidis* strain FAM18	1833	249
*N. meningitidis* strain alpha14	1782	255
*N. meningitidis* strain 53442	1881	234
*N. lactamica* strain 020-06	1873	92

^1^ Number of Correia Repeat Enclosed Elements in the genome (Table S9 to Table S16).

**Table 3:** Correia Repeat Enclosed Elements (CREE) associations with ncRNAs in *Neisseria* spp. genome sequences.

Strain	Intergenic Regions	SIPHT ncRNAs ^1^	CREE ^2^	within 1 kb ^3^	1 kb % ^4^	within 300 bp ^5^	300 bp % ^6^	Overlap ^7^	Overlap % ^8^
*N. gonorrhoeae* strain NCCP11945	2255	760	131	96	73%	86	66%	75	57%
*N. gonorrhoeae* strain FA1090	1806	976	123	100	81%	92	75%	73	59%
*N. meningitidis* strain MC58	2015	912	248	164	66%	131	53%	87	35%
*N. meningitidis* strain Z2491	1846	996	260	188	72%	151	58%	99	38%
*N. meningitidis* strain FAM18	1833	959	249	183	74%	143	57%	98	39%
*N. meningitidis* strain alpha14	1782	976	255	191	75%	147	58%	95	37%
*N. meningitidis* strain 53442	1881	846	234	151	65%	127	54%	85	36%
*N. lactamica* strain 020-06	1873	890	92	72	78%	59	64%	36	39%

^1^ Number of SIPHT predicted small non-coding RNAs in the genomes (Tables S1 to S8); ^2^ Number of Correia Repeat Enclosed Elements in the genome (Table S9 to Table S16); ^3^ CREE that have one or more SIPHT predicted ncRNAs within 1 kb (Table S17 to Table S24); ^4^ Percentage of CREE associated with predicted ncRNAs within 1 kb; ^5^ CREE that have one or more SIPHT predicted ncRNAs within 300 bp (Table S17 to Table S24); ^6^ Percentage of CREE associated with predicted ncRNA within 300 bp; ^7^ CREE that overlap one or more SIPHT predicted ncRNAs (Table S17 to Table S24); ^8^ Percentage of CREE that overlap with predicted ncRNAs.

## Supplementary Materials

The following are available online at www.mdpi.com/2076-2607/4/3/31/s1, Table S1: SIPHT predicted small non-coding RNAs in *Neisseria gonorrhoeae* strain NCCP11945, Table S2: SIPHT predicted small non-coding RNAs in *Neisseria gonorrhoeae* strain FA1090, Table S3: SIPHT predicted small non-coding RNAs in *Neisseria meningitidis* strain MC58, Table S4: SIPHT predicted small non-coding RNAs in *Neisseria meningitidis* strain Z2491, Table S5: SIPHT predicted small non-coding RNAs in *Neisseria meningitidis* strain FAM18, Table S6: SIPHT predicted small non-coding RNAs in *Neisseria meningitidis* strain alpha14, Table S7: SIPHT predicted small non-coding RNAs in *Neisseria meningitidis* strain 53442, Table S8: SIPHT predicted small non-coding RNAs in *Neisseria lactamica* strain 020-06, Table S9: CREE in *Neisseria gonorrhoeae* strain NCCP11945, Table S10: CREE in *Neisseria gonorrhoeae* strain FA1090, Table S11: CREE in *Neisseria meningitidis* strain MC58, Table S12: CREE in *Neisseria meningitidis* strain Z2491, Table S13: CREE in *Neisseria meningitidis* strain FAM18, Table S14: CREE in *Neisseria meningitidis* strain alpha14, Table S15: CREE in *Neisseria meningitidis* strain 53442, Table S16: CREE in *Neisseria lactamica* strain 020-06, Table S17: Association of CREE and ncRNAs in *Neisseria gonorrhoeae* strain NCCP11945, Table S18: Association of CREE and ncRNAs in *Neisseria gonorrhoeae* strain FA1090, Table S19: Association of CREE and ncRNAs in *Neisseria meningitidis* strain MC58, Table S20: Association of CREE and ncRNAs in *Neisseria meningitidis* strain Z2491, Table S21: Association of CREE and ncRNAs in *Neisseria meningitidis* strain FAM18, Table S22: Association of CREE and ncRNAs in *Neisseria meningitidis* strain alpha14, Table S23: Association of CREE and ncRNAs in *Neisseria meningitidis* strain 53442, Table S24: Association of CREE and ncRNAs in *Neisseria lactamica* strain 020-06, Table S25: Locations of the start or end points of the CREE and the closest predicted ncRNA in *Neisseria gonorrhoeae* strain NCCP11945, Table S26: RNA-seq data supporting candidacy of ncRNAs with nearby CREE in *Neisseria gonorrhoeae* strain NCCP11945, Table S27: Genes nearby to CREE that are nearby to ncRNAs in *Neisseria gonorrhoeae* strain NCCP11945.
